# Growth Factor–like Gene Regulation Is Separable from Survival and Maturation in Antibody-Secreting Cells

**DOI:** 10.4049/jimmunol.1801407

**Published:** 2019-01-14

**Authors:** Sophie Stephenson, Matthew A. Care, Im Fan, Alexandre Zougman, David R. Westhead, Gina M. Doody, Reuben M. Tooze

**Affiliations:** * Section of Experimental Haematology, Leeds Institute of Medical Research, St. James’s University Hospital, University of Leeds, Leeds LS9 7TF, United Kingdom;; † Bioinformatics Group, School of Molecular and Cellular Biology, University of Leeds, Leeds LS2 9JT, United Kingdom;; ‡ Haematological Malignancy Diagnostic Service, Leeds Teaching Hospitals National Health Service Trust, St. James’s University Hospital, Leeds LS9 7TF, United Kingdom; and; § Section of Biomarkers and Therapy, Leeds Institute of Cancer and Pathology, University of Leeds, Leeds LS9 7TF, United Kingdom

## Abstract

Recurrent mutational activation of the MAP kinase pathway in plasma cell myeloma implicates growth factor–like signaling responses in the biology of Ab-secreting cells (ASCs). Physiological ASCs survive in niche microenvironments, but how niche signals are propagated and integrated is poorly understood. In this study, we dissect such a response in human ASCs using an in vitro model. Applying time course expression data and parsimonious gene correlation network analysis (PGCNA), a new approach established by our group, we map expression changes that occur during the maturation of proliferating plasmablast to quiescent plasma cell under survival conditions including the potential niche signal TGF-β3. This analysis demonstrates a convergent pattern of differentiation, linking unfolded protein response/endoplasmic reticulum stress to secretory optimization, coordinated with cell cycle exit. TGF-β3 supports ASC survival while having a limited effect on gene expression including upregulation of CXCR4. This is associated with a significant shift in response to SDF1 in ASCs with amplified ERK1/2 activation, growth factor–like immediate early gene regulation and EGR1 protein expression. Similarly, ASCs responding to survival conditions initially induce partially overlapping sets of immediate early genes without sustaining the response. Thus, in human ASCs growth factor–like gene regulation is transiently imposed by niche signals but is not sustained during subsequent survival and maturation.

## Introduction

The generation and maintenance of functional Ab-secreting cells (ASCs) is essential to humoral immunity (1). Long-lived ASCs persist as plasma cells (PCs) in a range of different niche conditions in vivo, affording the potential to link sustained survival to phenotypic and functional diversity (2, 3). In PC neoplasia, abnormalities in the niche play a key role in sustaining the neoplastic clone (4), whereas targets of recurrent mutation in PC neoplasia identify pathways of potential functional importance to the biology of ASCs. One of the most frequently deregulated pathways is that of RAS/RAF/MAP kinase signaling (5).

Although it is widely accepted that PCs may exist in complex microenvironments across the spectrum of normal to neoplastic states, how the pattern of signals received by a PC may be integrated remains poorly understood. We and others have developed model systems allowing the generation and maintenance of long-lived human PCs in vitro, which provide tools to directly address this question in primary cells and link external cues to specific response pathways (6, 7). PCs are functionally defined as ASCs that have entered cell cycle quiescence and derive from a preceding proliferative ASC state referred to as plasmablasts (PBs). This transition is accompanied by phenotypic changes but is principally separated by entry into cell cycle quiescence (8). The ability of an ASC to survive as a PC can be conceptually reduced to the capacity of the cell to home to, reside in, and respond to relevant niche signals, and it has been argued that competition for niche residence may contribute to control of the long-lived PC pool (9, 10). The chemokine CXCL12/SDF1 has been identified as an important component of niche homing signals for PCs (11–13). Consequently, SDF1-rich mesenchymal stromal cells are considered to form an important element of the marrow niche (14, 15). In addition to secreting SDF1, bone marrow stromal cells have the capacity to secrete a diverse range of mediators among which is TGF-β (4). Cross-talk between TGF-β and SDF1 signaling pathways has been described in several cell systems (16, 17). Both pathways are involved in the process of epithelial–mesenchymal transition and hence with invasive and migratory behavior (18, 19). However, whether PCs integrate these signals and to what effect is not known.

Among the signaling pathways linked to SDF1 responses in lymphocytes is activation of the MAP kinase pathway (16, 20–22). Although the role of the MAP kinase pathway in normal PC biology is not defined, components of the pathway are recurrent targets of mutation in PC neoplasia including both upstream regulators such as the RAS oncogenes and downstream effector EGR1 (23–25). EGR1 mutation has been reported to show a particularly high cancer clonal fraction when mutated, suggesting that it may either exert a strong selective pressure or be an early event in pathogenesis (5). Interestingly, in a model of cell cycle progression established in human mammary epithelial cells, ERK-EGR1 signaling has been proposed to provide a threshold mechanism generating all-or-none decisions for cell cycle entry (26). Furthermore, EGR1 protein expression along with several other immediate early genes (IEGs) can act as a sensor for the duration of MAP kinase signaling (27–29).

In this paper, we analyze TGF-β3 and SDF1 responses in human PCs using time course expression data and network analysis. This provides evidence for a model of convergent differentiation largely independent of the conditions supporting PC survival during the transition from PB to quiescent PC state. SDF1 exposure provides a pulse of MAP kinase signaling, which can be significantly enhanced in the presence of TGF-β3. This translates into enhanced induction of IEG and EGR1 protein expression, mimicking classical growth factor responses. ASCs responding to in vitro survival conditions induce transient and partially overlapping sets of IEGs but without sustaining the response. These data indicate that ASCs responding to niche signals follow rules for growth factor–induced MAP kinase signaling established in model epithelial and neuronal cell lines, and that growth factor–like gene regulatory responses are a transient feature of the ASC response to niche signals.

## Materials and Methods

### Reagents

IL-2 (Miltenyi Biotec); IL-21, IL-6, IFN-α, TGF-β3, SDF1α, Osteoprotegerin, M-CSF, Osteopontin, SCF, MIF, IGF-BP2, IGF-II, and Activin A (PeproTech); Jagged-1, ANGPT1, VEGF-165 and VEGF-121 (R&D Systems); Multimeric-APRIL (Caltag); goat anti-human IgM & IgG F(ab′)_2_ fragments (Jackson ImmunoResearch); Hybridomax hybridoma growth supplement (Gentaur); Lipid Mixture 1, chemically defined (200×) and MEM Amino Acids Solution (50×) (Sigma-Aldrich); SB525334 (Selleckchem).

### Donors and cell isolation

Peripheral blood was obtained from healthy donors after informed consent. The number of donors per experiment is indicated in the figure legend. Mononuclear cells were isolated by Lymphoprep (Axis Shield) density gradient centrifugation. Total B cells were isolated by negative selection with the Memory B cell Isolation Kit (Miltenyi Biotec).

Bone marrow aspirates were from three anonymous donors and were derived from surplus clinical samples. Mononuclear cells were isolated by Lymphoprep and normal B cells and PCs identified by the gating strategy outlined in Supplemental Fig. 1A.

### Cell cultures

Cells were maintained in 24-well flat-bottom culture plates (Corning) and IMDM supplemented with Glutamax and 10% heat-inactivated FBS (Invitrogen), Hybridomax hybridoma growth supplement (11 μl/ml), lipid mixture 1, chemically defined, and MEM amino acids solution (both at 1× final concentration) from day 3 onwards.

#### Day 0 to day 3.

B cells were cultured at 2.5 × 10^5^/ml with IL-2 (20 U/ml), IL-21 (50 ng/ml), F(ab′)_2_ goat anti-human IgM & IgG (10 μg/ml) on γ-irradiated CD40L-expressing l cells (6.25 × 10^4^ per well).

#### Day 3 to day 6.

At day 3, cells were detached from the CD40L l cell layer and reseeded at 1 × 10^5^ per ml in media supplemented with IL-2 (20 U/ml) and IL-21 (50 ng/ml).

#### Day 6 onwards.

For cytokine combination experiments and TGF-β3 dose response, cells were seeded at 5 × 10^5^ per ml in media supplemented with IL-6 (10 ng/ml), IL-21 (50 ng/ml), IFN-α (100 U/ml), and combinations of Jagged 1 (2 μg/ml); Osteopontin (1 μg/ml); Osteoprotegerin, SCF, and IGF-BP2 (100 ng/ml); Activin A, and M-CSF (50 ng/ml); IGF-II (30 ng/ml): ANGPT1, VEGF-165, VEGF-121, MIF, and TGF-β3 (10 ng/ml or 0.1–1000 ng/ml for dose response). Cell culture was terminated at day 13.

For inhibitor experiments, cells were seeded at 5 × 10^5^ per ml in phenol red–free, serum-free IMDM (Invitrogen) for 18 h. Vehicle or SB525334 (1 μM) was added for 1 h, and then cells were stimulated with either IL-6 (10 ng/ml), IL-21 (50 ng/ml), and IFN-α (100 U/ml) or TGF-β3 (2.5 ng/ml) for 2 h.

For extended gene expression experiments, cells were reseeded at 1 × 10^6^ per ml in media supplemented with IL-6 (10 ng/ml), IL-21 (50 ng/ml), and either IFN-α (100 U/ml) or TGF-β3 (2.5 ng/ml) alone or in combination. Cells were refed at 3.5-d intervals and analyzed at indicated times.

For short gene expression experiments, cells were seeded at 1 × 10^6^ per ml in phenol red–free media supplemented with 0.5% heat-inactivated FBS, IL-6 (10 ng/ml), IL-21 (10 ng/ml) alone, or with TGF-β3 (2.5 ng/ml) for 20 h. SDF1 (1 ng/ml) was added, and cells analyzed at indicated times.

### Flow cytometric analysis and microscopy

Cells were analyzed using four- to six-color direct immunofluorescence staining on a BD LSR II (BD Biosciences) or Cytoflex S (Beckman Coulter) flow cytometer. The following Abs were used: CD19 PE (LT19), CD19 VioBlue (LT19), CD138 allophycocyanin (B-B4/44F9), and CD138 VioGreen (44F9) (Miltenyi Biotec); CD20 e450 (2H7) (eBioscience); CD27 FITC (M-T271), CD56 PECy7 (NCAM16.2), CD38 PECy7 (HB7), CD38 BUV395 (HB7), and phospho-Smad2/3 PE (BD Biosciences); CXCR4 PE, TGFBR2 (FAB241), and TGFBR3 (FAB242) (R&D Systems). Phosflow Lyse/Fix and Perm/Wash buffer (BD Biosciences) was used for the preparation of cells prior to Phosflow. Controls were isotype-matched Abs or FMOs. Dead cells were excluded by 7-AAD (BD Biosciences). Absolute cell counts were performed with CountBright beads (Invitrogen). Cell populations were gated on forward light scatter and side scatter profiles for viable cells determined independently in preliminary and parallel experiments. Analysis was performed with BD FACSDiva software 5.0 and FlowJo version 10 (BD Biosciences).

### RNA, cDNA, and RT-PCR

RNA was extracted with TRIzol (Invitrogen), subjected to DNAse I treatment (DNA-free Kit; Ambion) and reverse transcribed using Superscript II Reverse Transcriptase (Invitrogen). TaqMan Assays for *FOS* (Hs00170630_m1), *FOSB* (Hs00171851_m1), *EGR1* (Hs00152928_m1), and *PPP6C* (Hs00254827_m1) were carried out according to manufacturer’s instructions and run on a Stratagene Mx3005P.

### Protein analysis

At the indicated time points, cells were lysed in Laemmli buffer. Samples were separated by SDS-PAGE and transferred to nitrocellulose. Proteins were detected by ECL (SuperSignal West Pico PLUS or Femto Chemiluminescent substrate, Thermo Fisher Scientific) and visualized on a ChemiDoc (Bio-Rad) or film. Protein bands were quantitated using ImageJ software.

Abs used were β-ACTIN (Sigma-Aldrich); p-SMAD2 S465/467 (138D4), p-ERK1/2, ERK1/2, and EGR1 (Cell Signaling); goat anti-mouse HRP, goat anti-rabbit HRP (Jackson ImmunoResearch).

### Proteomics

Centrifuged and filtered (0.2 μm) supernatants and media were generated from duplicate samples of M2-10B4 cells either nonirradiated, irradiated, or irradiated and cultured in the presence of IL-6, IL-21, and IFN-α. STrap-based tryptic digestion was performed as previously described (30). Peptides were separated online by reversed-phase capillary liquid chromatography using EASY-nLC 1000 UPLC system (Thermo Fisher Scientific) connected to a capillary emitter column. LTQ Orbitrap Velos mass spectrometer (Thermo Fisher Scientific) was used for data acquisition. Raw data were processed against the Uniprot mouse protein sequence database (May 2013) using MaxQuant software (www.maxquant.org) (31). The maximum protein and peptide false discovery rates (FDRs) were set to 0.01.

### Immunohistochemistry

Surplus material from bone marrow trephine specimens assessed as reactive in diagnostic practice were stained with phospho-SMAD2 specific Ab (Cell Signaling rabbit polyclonal, catalog no. 3101). Staining was performed using DAKO Envision Flex and Detection System (Envision Flex+ high pH kit [K8002 DAKO]). Ag retrieval was carried out using heat-mediated Ag retrieval using pressure cooking for 6 min. DAKO Envision Flex+ kit was used with standard methods, Flex+ reagent with rabbit linker, DAB chromogen, and hematoxylin counterstain (complete protocol available on request). Stained slides were assessed on a Nikon Eclipse 80i microscope equipped with ×40 Plan Fluor objective and Nikon Digital Sight DS-Fi1 camera system.

### Expression data sets

Two gene expression data sets were generated, a long time course (LTC) and a short time course (STC). The LTC consists of a differentiation of B cells from four healthy donors from day 3 (activated B cell), day 6 (PB), and then posttreatment with three different conditions at day 6 +1, +3, +6, +12, +24 h (day 7), +48 h (day 8), +96 h (day 10), +168 h (day 13; PC), +336 h (day 20; PC). The three conditions were as follows: 1: (IL-6, IL-21, IFN-α), 2: (IL-6, IL-21, IFN-α, TGF-β3), and 3: (IL-6, IL-21, TGF-β3.). For each time there were four samples except for day 6 (biological replicates giving eight samples) and condition 3 (day 20), which only had one sample because of quality control. The STC consists of differentiating PBs (day 7) from three healthy donors maintained in low-serum media with or without TGF-β and then posttreatment with SDF-1 at +30, +120, +360 min.

### Gene expression data acquisition and analysis

Gene expression analysis was performed using HumanHT-12 v4 Expression BeadChips (Illumina) according to the manufacturer’s instructions and scanned with the Illumina BeadScanner. Initial data analysis was performed using GenomeStudio Gene Expression Module, and as previously described (M.A. Care, D.R. Westhead, and R.M. Tooze, manuscript posted on bioRxiv). Expression data sets for LTC and STC samples are available at https://www.ncbi.nlm.nih.gov/geo/query/acc.cgi with Gene Expression Omnibus accession number GSE120369.

### Gene signature data and enrichment analysis

A merged data set of 39,482 terms was generated from externally curated databases and gene ontology terms as previously described (Care, et al., manuscript posted on bioRxiv). Enrichment of gene lists for signatures was assessed using a hypergeometric test in which the draw is the gene list genes, the successes are the signature genes, and the population is the genes present on the platform.

### Parsimonious gene correlation network analysis

#### Probe selection.

Before probes were merged per gene the most informative probes were selected for the different time courses. For the LTC the probes differentially expressed between each condition at each time point and between day 3 and day 6 (*p* value <0.01; fold-change >1.2; *n* = 7073 probes) were merged with the probes σ^2^ > 0.05 (across median values per time point) giving a nonredundant set of 11,388 probes merged to 9063 genes. For the STC the probes differentially expressed between TGF-β^+/−^ at each time point and between each posttreatment time point and its corresponding pretreatment sample (*p* value <0.05; fold-change >1.2; *n* = 4471 probes) were merged with the probes σ^2^ > 0.05 (across median values per time point), giving a nonredundant set of 5302 probes, merged to 4705 genes.

#### Network analysis.

For details and validation of the parsimonious gene correlation network analysis (PGCNA) approach, see Care, et al. (manuscript posted on bioRxiv). In this paper, a brief description of the method will be given (see Fig. 3A for overview). After informative genes were selected, they were used to calculate Spearman rank correlations for all gene pairs using the Python scipy.stats package. For each gene (row) in a correlation matrix, only the three most correlated edges per gene were retained. The resulting matrix *M*, with entries written as *M* = (*m_ij_*), was made symmetrical by setting *m_ij_ = m_ji_* for all indices *i* and *j* so that *M* = M^T^ (its transpose). This reduced the edges in LTC from 41,064,453 to 24,683 and in STC from 11,066,160 to 11,458. The correlation matrices were clustered 10,000 using the fast unfolding of communities in large networks algorithm (version 0.3) and the 100 best (judged by modularity score) were used for downstream analysis. The most informative clustering was selected using gene signature enrichment visualized using the Gephi package (version 0.82) with the ForceAtlas2 layout method and an interactive HTML5 web visualization exported using the σ.js library (https://github.com/oxfordinternetinstitute/gephi-plugins/tree/sigmaexporter-plugin) (32, 33).

#### Network availability.

Interactive networks and metadata are available at http://pgcna-tgfb.gets-it.net.

#### Module overlaps.

The overlap of the modules between the networks at the gene and signature level was assessed using a hypergeometric test and the overlap visualized as a Python matplotlib heatmap of −log_10_ *p* values. The signatures were prefiltered to *p* value <0.001 and ≥5 and ≤1000 genes.

#### Heatmap visualizations.

Visualizations of the clustered gene expression data and enriched gene signatures were carried out using the Broad GENE-E package (https://software.broadinstitute.org/GENE-E/). Data were hierarchically clustered (Pearson correlations and average linkage). For the visualizations of gene signature enrichments, the signatures were filtered to FDR <0.1 and ≥5 and ≤1500 genes for the signature sets, selecting the top 30 most significant signatures per module, excluding signature sets MSigDB_C7 and GeneSigDB.

### Ethical approval

Approval for this study was provided by U.K. National Research Ethics Service via the Leeds East Research Ethics Committee, approval reference 07/Q1206/47.

## Results

### TGF-β supports in vitro PC survival

**FIGURE 1.**
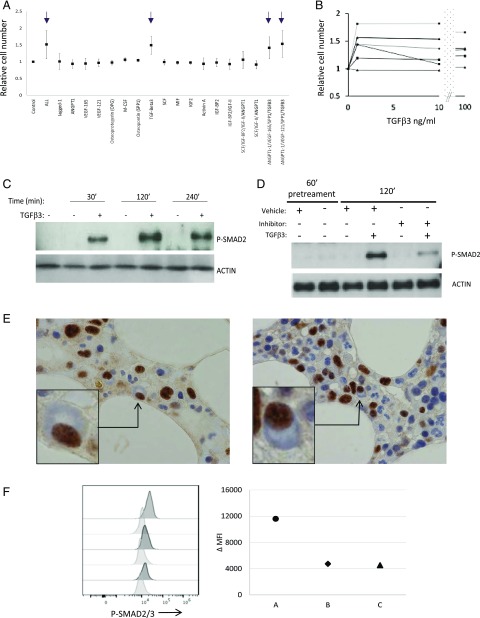
TGF-β3 provides a potential survival signal for ASCs. (**A**) A series of proteins, identified from the secretome of stromal cells, was tested for the capacity to promote PC survival in combination with IL-6 and IL-21. Data are shown as average fold cell number relative to IL-6 and IL-21 alone (control) from a total of four donors in two independent experiments. Proteins tested are indicated on the *x*-axis; arrows identify all conditions in which TGF-β3 was present. (**B**) Dose–response data of TGF-β3 in nanogram per milliliter (*x*-axis), showing viable PC number at day 13 of culture (*y*-axis) for five donors. (**C**) Time course of SMAD2 phosphorylation induced by TGF-β3 stimulation (2 ng/ml) at day 6 relative to control, representative time points indicated above the Western blot. (**D**) Inhibition of SMAD2 phosphorylation with inhibitor SB525334. Shown is induced SMAD2 phosphorylation at 120 min in ASCs in the presence or absence of TGF-β3, either pretreated or not pretreated with inhibitor for 60 min. (**E**) Immunohistochemical detection of phosphorylated SMAD2 in normal human bone marrow trephine samples. Two representative fields are shown with morphologically defined PCs identified with black arrows and shown in cropped insert to illustrate morphology of a phospho-SMAD2/3 positive PC. (**F**) Intracellular phospho-SMAD2/3 staining in PCs gated on CD19 expressing PCs showing representative example and the change in mean fluorescence intensity (ΔMFI) from three independent bone marrow samples. Isotype control pale gray, phospho-SMAD2/3 dark gray. Original magnification ×40.

PC survival can be supported in vitro in a contact-independent fashion by stromal cell lines (6). Among the secreted factors released by such cell lines are SDF1 and members of the TGF-β family, and in the case of the M2-10B4 cell line, which efficiently supports PC survival in our model system, this is TGF-β3 (Supplemental Table I). Indeed, among a series of recombinant factors tested, TGF-β3 acted in conjunction with IL-6 to support PC survival in vitro, independent of stromal support (Fig. 1A, 1B). Upon TGF-β3 stimulation, PB ASCs at day 9 of culture induced robust SMAD2 phosphorylation, which was sustained for at least 4 h (Fig. 1C). This SMAD2 phosphorylation could be inhibited by pretreatment with a TGF-β receptor inhibitor consistent with canonical signaling (Fig. 1D). Immunohistochemical staining of bone marrow sections identified evidence of nuclear phospho-SMAD2 in PCs (Fig. 1E) (34), which was corroborated by flow cytometric analysis of ex vivo bone marrow PCs (Fig. 1F, Supplemental Fig. 1A). Thus, phosphorylation of SMAD2 can be observed as a feature of bone marrow PCs, and TGF-β provides a potential niche signal capable of driving SMAD2 phosphorylation in primary human ASCs in vitro.

### TGF-β supports execution of the terminal phase of PC differentiation

The sustained activation of SMAD2 phosphorylation observed following PB stimulation with TGF-β3 suggested the potential capacity to impact on subsequent gene expression in PCs. We therefore performed a gene expression time course experiment focusing on the differentiation window of PBs to PCs across sequential time points between day 6 and day 20 of culture in the presence of TGF-β3 or IFN-α, which represents our previous standard cytokine context (3) or both TGF-β3 and IFN-α. The overall phenotypic maturation of PBs to PCs proceeded without significant differences between the three conditions to day 20 (Supplemental Fig. 1B); TGFBR2 and TGFBR3 surface expression was highest prior to TGF-β3 exposure but remained detectable in mature PCs maintained in TGF-β3 (Supplemental Fig. 1C). The numbers of surviving cells at each time point showed no significant difference although exhibiting a trend toward better survival in the presence of IFN-α (Supplemental Fig. 1D).

**FIGURE 2.**
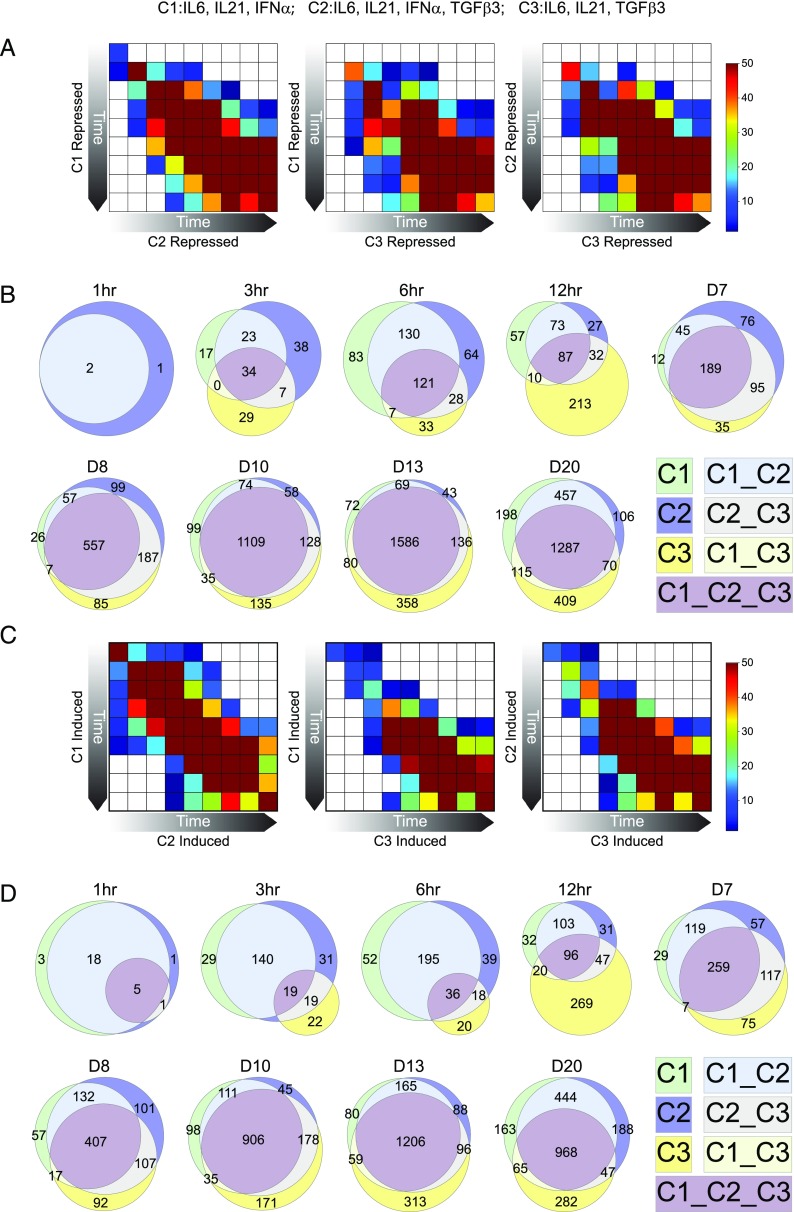
Maturation of PBs to PCs is associated with convergent patterns of gene induction and repression. Genes differentially expressed in each of the three conditions (C1: IFN-α, C2: IFN-α/TGF-β3, C3: TGF-β3, all with IL-6 and IL-21) at each time point of the time course was determined relative to the initial gene expression at the PB state. The overlap of differentially expressed genes at each time point was then assessed in pairwise fashion and displayed as a heatmap (**A**) repressed and (**C**) induced, with the significance of overlap as −log10 *p* values displayed as a color scale, as indicated at the far right of (A) and (C) (white not significant, dark blue *p* value <0.05, to brown highly significant overlap). By comparing the overlap of any given time point to all others in the series, the display highlights the overall coordinated progression of differential expression between the conditions. The conditions are indicated on the *x*- and *y*-axis labels for each comparison: left panel (C1 *y*-axis versus C2 *x*-axis), middle panel (C1 *y*-axis versus C3 *x*-axis), and right panel (C2 *y*-axis versus C3 *x*-axis). (**B** and **D**) represent the overlaps of differentially repressed and induced genes, respectively, at each time point, with each condition and intersect color coded as indicated in the figure. The size of the three-way intersect illustrates the progression toward a common set of regulated genes relative to genes uniquely regulated in each condition.

To analyze the gene expression data (Supplemental Table II) we first considered differential gene expression, comparing each time/condition pair against the day 6 PB state at which the different cytokine conditions were added. Considering genes with fold-change >1.5 and FDR-corrected *p* value <0.05, we assessed the extent of overlap between sets of repressed and induced genes (Supplemental Table III) under each condition in all-by-all pairwise comparisons (Fig. 2A, 2C) and as overlapping Venn diagrams (Fig. 2B, 2D). The pairwise comparison heatmaps assess how the sets of significantly downregulated (repressed) or significantly upregulated (induced) genes at a given time point and condition relate to all the sets of significantly upregulated or downregulated genes in either of the other two conditions. This visualization emphasizes the progressive convergence of differentially expressed genes with time. Consistent with our previous analysis (3), a group of differentially up- and downregulated genes are characteristic of the response to IFN-α (C1 and C2) and are induced within 3 h of stimulation regardless of the presence or absence of TGF-β3. Thus, the two conditions of differentiation in the presence of IFN-α (C1 and C2) are more closely related at earlier time points, particularly for induced genes. Nonetheless, from 24 h (day 7) onward, there is convergence onto a common set of induced and repressed genes irrespective of the cytokine condition. Venn diagrams assessed the extent of overlap at each time point as a three-way comparison, again illustrating convergence onto a common set of induced and repressed genes over time. However, this also allowed the representation of genes that were unique to each condition, identifying a subset of genes regulated in TGF-β3 conditions (Fig. 2C, 2D).

Thus, the time course supports a pattern of convergent gene regulation during the PB to PC transition, whereby irrespective of the culture conditions allowing PC survival, a common set of induced and repressed genes emerges. Overlaid onto this are groups of differentially expressed genes that are specific to the stimuli that are permissive to survival and maturation.

### PGCNA resolves discrete biological modules of gene expression

**FIGURE 3.**
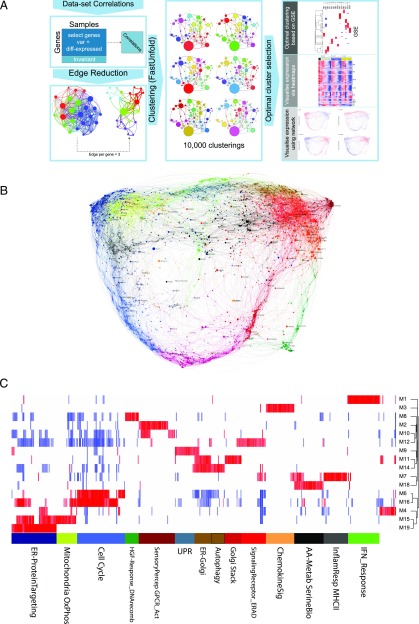
Application of PGCNA to time course gene expression data of PB to PC transition. (**A**) Outline of PGCNA approach as detailed in *Materials and Methods* and Care, et al. (manuscript posted on bioRxiv) (**B**) Network representation of the modular pattern of gene expression during the transition of PB to PC. Network modules are color coded; for interactive version go to http://pgcna-tgfb.gets-it.net/. (**C**) Heatmap summary representation of gene ontology and signature separation between network modules (filtered FDR <0.1 and ≥5 and ≤1500 genes, selecting the top 30 most significant signatures per module). Significant enrichment or depletion illustrated on red/blue scale, *x*-axis (signatures), and *y*-axis (modules). Hierarchical clustering according to gene signature enrichment. Indicative module terms shown below. For high-resolution version and extended data see Supplemental Fig. 3 and Supplemental Table V.

To explore the biology associated with differential gene expression during the PB to PC transition, we applied an expression networking approach, which we refer to as PGCNA (Fig. 3A). In parallel work, we extensively validate the utility of this method for evaluation of primary cancer expression data sets (M.A. Care, D.R. Westhead, and R.M. Tooze, manuscript posted on bioRxiv). In this study, we test its utility in analyzing time course expression data.

**FIGURE 4.**
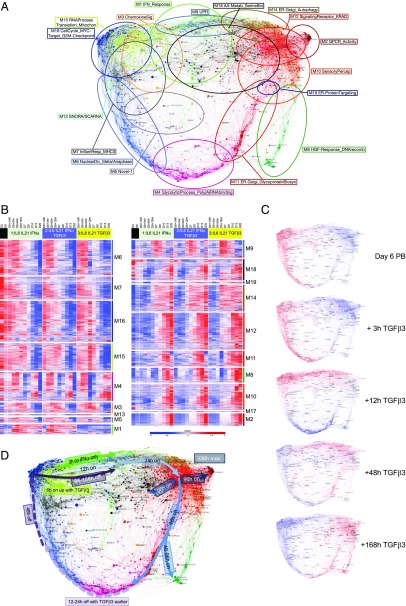
Dynamics of gene expression during PB to PC transition. (**A**) Module summary terms identify different aspects of cell biology represented across the expression network. (**B**) Heatmap displaying the pattern of gene expression across the time course for the three conditions (black, day 6 reference; light green, IL-6/IL-21/IFN-α; blue, IL-6/IL-21/IFN-α/TGF-β3; yellow, IL-6/IL-21/TGF-β3): module numbers indicated on the right, *z*-score gene expression, blue (−3 low) and red (+3 high): color scale as indicated, right lower edge. Showing the median expression across four donors per timepoint, except day 20 where quality control failure reduced donor numbers. (**C**) Overlay of gene expression *z*-scores for all genes in the network shown in blue (low) to red (high) *z*-score color scale. Day 6 provides the starting reference point for the sequential expression patterns observed under C3: IL-6/IL-21/TGF-β3 condition at the time points indicated to the right of each network image. For interactive versions of all networks go to http://pgcna-tgfb.gets-it.net/. (**D**) Summary of gene expression flow across the time course illustrated with arrows in the network. Induced expression in solid lines, repression in dotted lines, with relevant time points and conditions indicated in the figure.

The resulting expression network for the transition of PBs into PCs divides into 19 modules (Fig. 3B, Supplemental Table IV and online resource). The separation of specific functions and ontologies between network modules was assessed using enrichment of signature and ontology terms across each module of the network (Fig. 3C, Supplemental Fig. 2, Supplemental Table V). The summary display of signature and ontology term enrichments illustrates the resolution of biological processes between modules as discrete bars of enrichment (red) and depletion (blue). These modules in turn reflect differential regulation of these processes across the time course of differentiation (Fig. 4). The initial step of the differentiation of PBs into quiescent PCs is accompanied within 3 h by an IFN response module (M1_IFNResponse) where this cytokine is present, and this remains, in part, superimposed on the overall network of gene expression of the differentiating PC at all time points in conditions containing IFN-α (Fig. 4B, Supplemental Fig. 3) (3). TGF-β3 effects are more distributed within the network, including genes in a module linked to both metabolic and translational processes as well as genes linked to the B cell state and the chemokine receptor gene *CXCR4* (M15_RNAProcessing Translation_Mitochondrion_OxPhos).

Following the initial response to cytokine conditions, a module linked to genes regulated by IRE1 and the unfolded protein response (UPR) and overlapping with endoplasmic reticulum (ER)/Golgi components is upregulated (M9_UPR) (Fig. 4B, 4C, Supplemental Fig. 3). This initial ER response is relatively accelerated in the presence of TGF-β3, with greatest expression at 12 h rather than 24 h. In all conditions, this is followed by sequential modules of gene expression, which separate secretory pathway components and genes linked with autophagy (M14_ERGolgi_Autophagy) from those enriched for ER-associated protein degradation (ERAD) and ER quality control pathways (M12_SignalingReceptor_ERAD) and those linked to the Golgi apparatus and glycoprotein biosynthesis (M11_ER-Golgi_GlycoproteinBiosyn). Thus, as ASCs progress from PB to the PC state, modules of genes indicative of an ER stress response precede those linked to optimization of secretory activity.

The induction of ER-related modules is accompanied, in contrast, by progressive gene silencing. This initiates with the repression of genes linked to a subset of cell cycle–related processes, in particular those related to the mitotic anaphase and sister chromatid segregation at 12–24 h (M6_NuclearDivision_Meta/Anaphase). Other components of the cell cycle remain expressed up to 24 h after transition into PC-supportive conditions (M16_CellCycle_MYCTarget_G2MCheckpoint). The initial extinction of cell cycle–related components is modestly accelerated in the context of TGF-β3 (Fig. 4B (M6 & M16), Supplemental Fig. 3).

Subsequently, from 48 h onward, the main cell cycle and DNA replication module (M16_CellCycle_MYCTarget_G2MCheckpoint) and a module linked to Ag presentation via MHC class II and inflammatory signaling (M7_InflammatoryResponse_MHCII) are repressed. This is followed by the eventual repression of the UPR gene module (M9_UPR), initially induced at the start of the transition to the PC state.

The late phase, beyond 96 h (day 10), is accompanied by the progressive concentration of PC-related gene expression into secretory and alternate metabolic-related modules, including those linked to amino acid metabolism (M18_AminoAcidMetab_SerineBiosyn). Distinctively mature PC-associated modules are enriched for microRNA and noncoding RNA genes as well as subsets of signaling receptors including G protein–coupled receptors (M2_GPCR_Activity).

Regulation of BCL2 family members has been identified as an important element of PC survival (35). Among this gene family, both pro- and anti-apoptotic members are dispersed across several modules of the network. Notable patterns for anti-apoptotic family members are provided by *BCL2A1*, which appears as a node in module M3_ChemokineSignaling, reflecting a modest transient wave of upregulation during the initial 48 h after transition into PC conditions, whereas *BCL2*, *BCL2L1* (*BCLX*), and *MCL1* belong, respectively, in M18_AA-Metabolism_Serinebiosynthesis, M12_SignalingReceptor_ERAD, and M11_ER-Golgi_GlycoproteinBioSynthesis, reflecting patterns of sustained upregulation at later stages of PC differentiation (Supplemental Fig. 4A). Among proapoptotic family members, *BIK* and *BCL2L11* (*BIM*) show a pattern of acute upregulation over the initial 48 h after transfer into PB maturation conditions. By contrast, expression of proapoptotic family members *BMF* and *BBC3* (*PUMA*) show more sustained induction and belong to modules M12_SignalingReceptor_ERAD and M2_GPCR_Activity, respectively, the latter being distinctively associated with the latest stages of PC differentiation (Supplemental Fig. 4B). These data at mRNA level support a common pattern largely invariant in relation to the tested conditions promoting PC survival, with progressive expression of *BMF* and *BBC3 (PUMA)* as primary proapoptotic effectors, offset by concerted upregulation of *MCL1*, *BCL2*, and *BCL2L1 (BCLX*) in mature steady-state PCs.

This network analysis of the PB to PC transition thus provides a refined view of the final steps of the differentiation of ASCs, linking gene expression related to secretory optimization to cell cycle exit and expression of select surface receptors. Exposure to TGF-β3, unlike IFN-α, did not impose a distinct single module of gene expression in the conditions necessary for this long-term time course, instead exerting more distributed effects across several modules.

### TGF-β modulates SDF1 signaling to the ERK MAP kinase pathway

**FIGURE 5.**
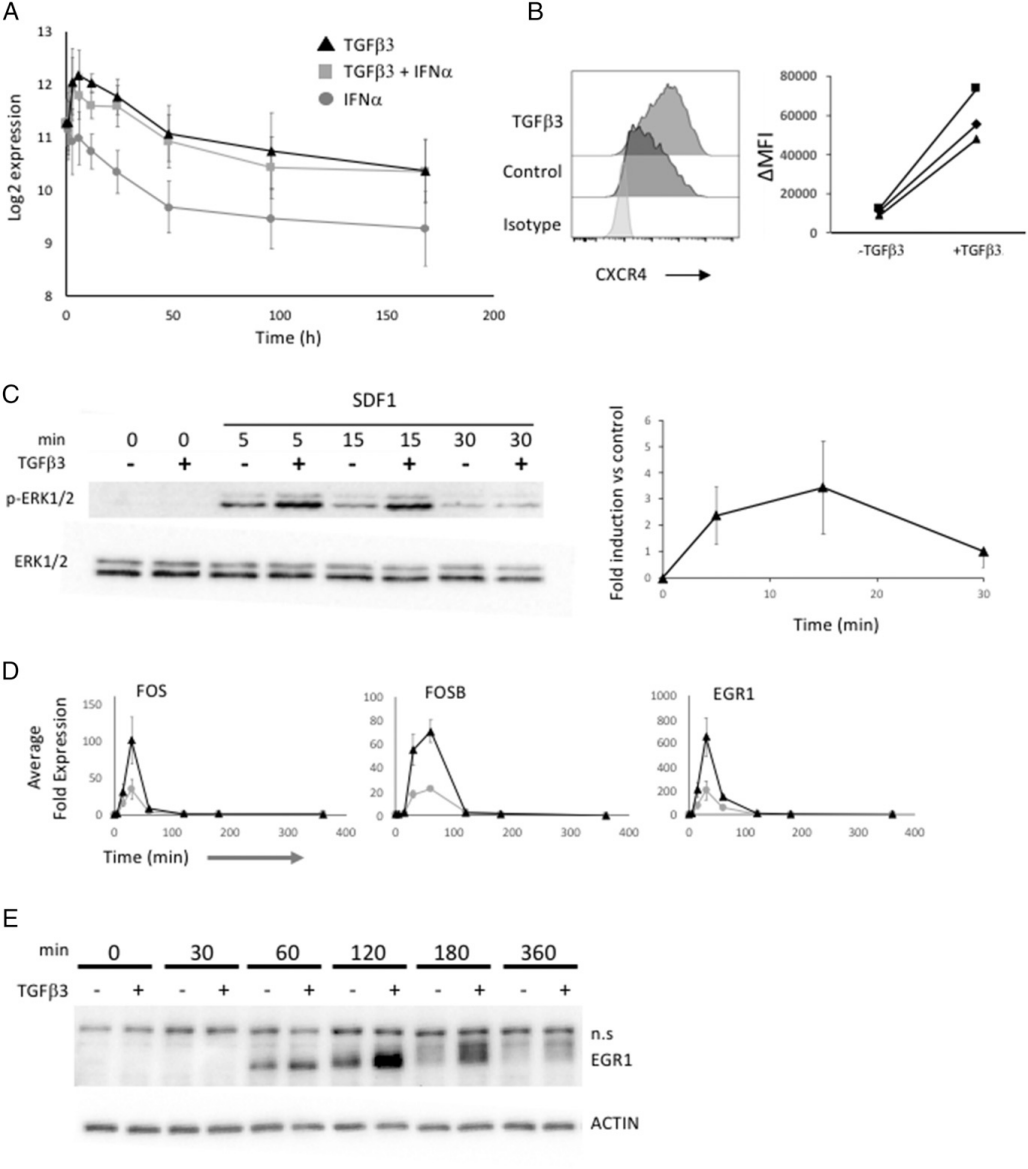
TGF-β3 induces CXCR4 expression in ASCs. (**A**) Expression of *CXCR4* mRNA across B cell differentiation showing average and SD (*n* = 4 samples per time point and condition) of log-2 gene expression values for the three conditions TGF-β3 (black triangle), TGF-β3 + IFN-α (gray square), and IFN-α (gray circle), each in the presence of IL-6 and IL-21. (**B**) Representative data for CXCR4 surface expression on ASCs at day 7 of culture in the presence (mid gray upper contour) or absence (dark gray middle contour) of TGF-β3 relative to isotype control light gray (lower contour) in control-treated samples (light gray). Summary of change in CXCR4 mean fluorescence intensity (ΔMFI) for three representative donors. (**C**) Time course of ERK phosphorylation induced by SDF1 treatment from 0 to 30 min in the presence (+) or absence (−) of prior TGF-β3 exposure as indicated above the blot: upper panel Western blot for phospho-ERK1/2, lower panel Western blot for total ERK1/2. Quantitation of fold ERK1/2 phosphorylation across the time course of TGF-β3–treated versus untreated control samples (three independent replicates). (**D**) Quantitation of *FOS*, *FOSB*, and *EGR1* mRNA expression following SDF1 treatment of ASCs in the presence (black triangle) or absence (gray circle) of prior TGF-β treatment, average, and SE of mean from three replicates. (**E**) EGR1 protein expression across the indicated time course following SDF1 treatment in ASCs in the presence (+) or absence (−) of prior TGF-β3 treatment as indicated. Lower panel ACTIN loading control.

Among the genes differentially expressed in response to TGF-β3 at the early phase of the response and subsequently sustained was *CXCR4* (Fig. 5A), which is a known target of TGF-β signaling in other cellular contexts (16, 17). Because SDF1-CXCR4 signaling is considered to be a primary determinant of recruitment and residence of PCs in the bone marrow niche (11–13), we focused further on this aspect of the response.

CXCR4 is expressed on the surface of PBs and PCs and was increased on PBs following treatment with TGF-β3 (Fig. 5B). SDF1 signaling via CXCR4 can mediate a variety of intracellular signaling events. Among these is activation of the MAP kinase pathway (21, 36). Indeed, treatment of ASCs with SDF1 at day 7 of culture led to rapid induction of ERK1/2 phosphorylation (Fig. 5C). Furthermore ERK1/2 phosphorylation induced in response to SDF1 was amplified and sustained by pretreatment with TGF-β3 (Fig. 5C). Receptor density has been identified as a means by which the MAP kinase pathway output from growth factor receptors can be modified (27, 29), and these data would be consistent with such a model.

To evaluate whether the enhanced ERK1/2 activation impacted on gene regulation, we examined the expression of IEGs *EGR1*, *FOS*, and *FOSB*. Following SDF1 stimulation, PBs exhibited a rapid induction of *FOS* and *EGR1* at 15 and 30 min, with decay by 60 min and return to near baseline by 120 min (Fig. 5D). *FOSB* showed a slightly delayed kinetics with initial upregulation at 30 min and peak at 60 min. For each of these genes, pretreatment with TGF-β3 substantially increased the amplitude of the response in all donors, albeit with variability of the absolute values between donors. Thus, the primed state of such immediate early response genes is retained in PBs as at other stages of the B cell lineage (37).

EGR1 is a transcriptional regulator implicated in PC biology through its recurrent mutation in PC myeloma (23–25). Furthermore, EGR1 provides a potential sensor of the duration of MAP kinase signaling, whereby persistence of ERK1/2 activation allows phosphorylation of EGR1, stabilizing its expression and impacting on downstream function (27). We therefore examined the expression of EGR1 at protein level following SDF1 treatment in the presence or absence of prior TGF-β3 exposure. SDF1 treatment, without prior TGF-β3 exposure, induced expression of EGR1 protein, which was detectable by 60 min, peaked at 120 min, and returned to near baseline at 180 min. By contrast, SDF1 treatment following prior TGF-β3 exposure substantially increased the magnitude of EGR1 protein expression at both 120 and 180 min. EGR1 protein expression remained significantly above baseline at 360 min (Fig. 5E). This impact on EGR1 expression was linked to an altered mobility in the EGR1 band, which is consistent with the effects of phosphorylation mediated by persistent ERK1/2 activation (27).

Thus, EGR1 is dynamically regulated in response to SDF1 in primary human ASCs. The pattern of regulation is consistent with a mechanism whereby enhanced and sustained ERK signaling is linked both to the regulation of the magnitude of gene expression and to subsequent protein phosphorylation and stabilization (28, 29).

### SDF1 induces a growth factor–like gene regulatory response in ASCs

To provide a more global picture of the impact of SDF1 signaling in ASCs in the presence or absence of TGF-β3 we again used a gene expression time course approach. Differentiating PBs were maintained in low-serum media, with or without TGF-β3 for 20 h prior to acute stimulation with SDF1. The response was sampled at baseline immediately prior to SDF1 exposure and 30, 120, and 360 min after stimulation to catch the dynamics of immediately early and delayed response gene expression (Supplemental Table VI) (38).

For this analysis we initially considered differentially expressed genes with FDR-corrected *p* value <0.05 (Supplemental Table VII). After 20 h TGF-β3 treatment, 12 genes were significantly upregulated and five genes significantly downregulated relative to the control-treated sample. Among these was *CXCR4*, which remained significantly differentially expressed at all times point of the experiment. Following addition of SDF1, a pulse of differentially expressed genes was observed, which was broader in the presence (33 genes significantly induced) than in the absence of TGF-β3 (14 genes significantly induced) relative to baseline for the respective condition at 30 min. All but one of the 14 genes significantly induced following SDF1 treatment in the absence of TGF-β3 were encompassed in the 33 genes induced in the presence of TGF-β3; this included *EGR1*, *FOS*, and *FOSB* as well as *ATF3*, *EGR2*, *CD69*, and *KLF6*. In the presence of TGF-β3 additional genes significantly upregulated included *JUNB*, *MIR155HG*, and *SRF*. At 120 min after SDF1 treatment in the presence or absence of TGF-β3, 154, and 44 genes were significantly upregulated, and 172 and 18 genes significantly downregulated, respectively. Again, the majority of the genes regulated in the absence of TGF-β3 were included among genes regulated in the presence of TGF-β3, consistent with a substantially diversified response in cells exposed to TGF-β3. In both conditions, by 360 min after SDF1 stimulation, the response was curtailed.

**FIGURE 6.**
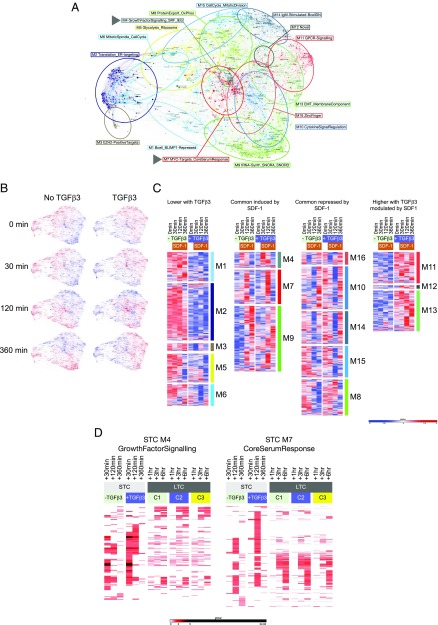
Network analysis of SDF1-induced gene expression in ASCs. (**A**) PGCNA analysis of gene expression derived from a STC following SDF1 treatment in presence or absence of TGF-β3. Network comprises 16 modules; signature enrichment analysis was used to determine biological features of individual modules and derive representative summary terms. Arrow heads identify modules M4 (GrowthFactorSignaling) and M7 (CoreSerumResponse). (**B**) Overlay of gene expression *z*-scores for genes in the network shown in blue (low) to red (high) *z*-score color scale. Conditions are indicated above the network images with time point of sampling in relation to SDF1 treatment to the side (for interactive networks see go to http://pgcna-tgfb.gets-it.net/; detailed version Supplemental Fig. 5). (**C**) Module expression values illustrated as heatmap separated into categories of lower with TGF-β, induced following SDF1, repressed following SDF1 and higher with TGF-β. Showing the median expression across three donors per timepoint. (**D**) Heatmap illustrating dynamics of M4 and M7 gene expression derived from the STC and comparing patterns in the STC data to the LTC data with the relevant conditions C1, C2, and C3 indicated in the figure. Shows expression fold change for each gene relative to baseline (STC: 0 min per day 7, LTC: day 6).

We next considered the wider pattern of gene expression change induced in this model using PGCNA and a lenient threshold for differential expression. This resulted in a network comprised of 16 modules (Fig. 6A, Supplemental Table VIII, and online resource). Gene ontology and signature enrichment analysis indicated that these modules were associated with coherent biology, suggesting that additional insight into the overall response could be derived by integrating subtle changes across many genes in this manner (Supplemental Fig. 5, Supplemental Table IX).

Prior to SDF1 exposure, modules of coexpressed genes differentiated the baseline states with or without TGF-β3 (Fig. 6B, 6C, Supplemental Fig. 6). Cells cultured in the absence of TGF-β3 showed generally higher expression of several modules, including one enriched for BLIMP1 target genes (M1_Bcell_BLIMP1Repressed), genes involved in translation (M2_Translation_ER-Targeting), and genes linked to the mitotic spindle and cell cycle (M6_MitoticSpindle_CellCycle). By contrast, cells cultured in the presence of TGF-β3 for 20 h showed relative upregulation of gene modules linked to GPCR signaling (M11_GPCR-Signaling) and integral membrane proteins (M13_EMT_MembraneComponent), including *CXCR4*. Thus, TGF-β3 exposure for 20 h promoted a subtle shift toward a more differentiated phenotype in the ASC population. Cells under both conditions, prior to SDF1 exposure, retained elements of cell cycle–associated gene expression (M15_CellCycle_MitoticDivision) and expression of genes linked to protein export and oxidative phosphorylation (M8_ProteinExport_OxPhos).

Against this backdrop, PGCNA indicated a coordinated response of up- and downregulated gene expression following SDF1 treatment. At 30 min this was characterized by the induced expression of genes focused in a single module enriched for characteristics of growth factor responses, targets of serum response factor, and IEGs (M4_GrowthFactorSignaling_SRF_IEG) (Fig. 6B, 6C, Supplemental Fig. 6). A second module of delayed responses genes was induced at the 120 min time point, again with greater magnitude in the TGF-β3–treated cells, which was enriched for core serum response–associated genes (M7_MYC-Targets_CoreSerumResponse). A further module (M9_tRNA-Synth_SNORA_SNORD) was upregulated at 360 min, consistent with the kinetics of secondary response genes, and included genes associated with amino acyl tRNA synthesis, SNORA/SNORD genes and the antioxidant genes *HMOX1*, *NQO1*, and *GCLM*. The sequence of common upregulated modules was also paralleled in modules of sequential gene repression following SDF1 exposure at 30 and 120 min with subsequent re-expression.

The analysis with PGCNA thus emphasized a sequence of gene expression changes following SDF1 exposure, with strong parallels to that of growth factor responses, indicating that such a regulatory response can be driven by this niche signal in ASCs. To assess whether induction of a growth factor–like signaling module was a unique feature of the SDF1 response, we re-examined the LTC data set focusing on expression of genes belonging to the growth factor response and core serum response modules induced by SDF1 (M4 and M7) (Fig. 6D). Although a core component of IEGs, including *FOS, FOSB, JUN, EGR1, EGR2*, and *EGR3* were acutely induced by signals promoting survival, this was to a more modest degree than observed in response to SDF1 and did not extend across the M4 module genes. In contrast, a larger proportion of the M7 module genes showed related patterns of regulation at +3 h and beyond. We conclude that a pulse of growth factor–like signaling can be delivered in ASCs, in particular exemplified by the acute response to SDF1, but that this response is not an intrinsic requirement for PC survival, at least as assessed in vitro.

## Discussion

The data presented in this paper support the conclusion that TGF-β can act as part of a realized in vitro PC niche and in this context can mediate cross-talk with the SDF1-CXCR4 pathway. The demonstration that TGF-β stimulation alters expression of CXCR4 and the nature of signaling responses to SDF1 is shared with other cell types (16, 17). In this paper, we have dissected this response in ASCs in detail and identified EGR1 and IEGs as a proximal point of signal integration upstream of subsequent waves of gene expression. Furthermore, by comparing the modular patterns of gene expression induced during cultures supporting PC survival and those following acute SDF1 exposure, we find that growth factor–like gene regulation is separable from sustained gene expression associated with ASC survival/maturation.

The data additionally provide insight into the sequence of gene expression changes during the final stage of human ASC maturation from the PB to the PC state. To address this question, we have applied a method we have developed, which we refer to as PGCNA, to gene expression time course data. PGCNA resolves a detailed sequence of expression modules that are both induced and repressed as the ASC completes its maturation to the quiescent PC state. In the context of our LTC data set covering maturation to the PC state, this illustrates a wave of UPR-related gene expression as an initial feature of the transition from PB to PC state. This is eventually extinguished but precedes the upregulation of several distinct modules of secretory pathway–related gene expression. This is consistent with a model in which the optimal adaptation for secretory capacity is not completed at the phenotypically defined PB ASC stage and that a classical UPR accompanies the final stages of differentiation to the quiescent PC state. Indeed, in murine PC differentiation, the UPR transcription factor XBP1 is largely dispensable for the earlier phenotypic maturation but is essential for the optimization of PCs for maximal secretory activity (39).

In relation to TGF-β signaling, our data illustrate that classical SMAD phosphorylation is readily activated and sustained in ASCs but that the overall effects on gene expression are modest. Low serum presents a limiting factor for long-term PC cultures, but in short-term cultures this is not the case, and in this context, analysis of subtle gene expression changes with PGCNA indicates that TGF-β3 treatment accelerated extinction of BLIMP1 target genes and elements of the cell cycle. These features are consistent with the established role of TGF-β as a factor capable of contributing to the establishment of cell cycle quiescence in other systems (40). At earlier stages of B cell activation, TGF-β, and TGF-β3 in particular, may act to impair B cell activation and commitment to differentiation (41). However, our data indicate that the effect of TGF-β is different when this signal is encountered by a B cell already committed to differentiation and at the plasmablast stage. In this context, TGF-β signals enhance ASC survival and facilitate the completion of differentiation to the plasma cell stage.

In addition to stromal cells, several potential sources of TGF-β exist in the bone marrow. In the context of the hematopoietic stem cell niche, characterized sources include Schwann cells and megakaryocytes (42–45), and the latter have previously been implicated as contributors to the PC niche (46). Recently, bone marrow–resident regulatory T (Treg) cells have been implicated in the support of bone marrow PCs (47). Similarly, bone marrow Treg cells localize in proximity to perivascular stromal cells and contribute to maintenance of hematopoietic stem cell quiescence (48). Such cells may provide a further source of TGF-β. Indeed, in mice subsets of LAG3^+^, Treg cells can provide a significant source of TGF-β3 (49), although whether this subset can contribute to the bone marrow Treg cell pool in mouse or human is, to our knowledge, undefined. Thus, there is evidence to suggests several possible sources of TGF-β signals that can colocalize with sources of SDF1 in bone marrow niche environments. Defining the principal sources of TGF-β relevant for bone marrow PCs and how these may contribute to the PC niche in different marrow states provides an interesting avenue for future investigation.

The SDF1–CXCR4 axis represents a primary determinant of PC niche homing (11–13). Although our analysis has focused on the interplay between TGF-β and SDF1 signaling in relation to transcriptional response in ASCs, in terms of ASC migration, different scenarios could be envisaged in which the enhanced transcriptional response might either remain coupled with or be uncoupled from SDF1-induced migration. Fine tuning of CXCR4 responses is critical for normal ASC localization, as illustrated by the effects of mutations in CXCR4 in mice that copy the gain-of-function mutations observed in warts-hypogammaglobulinemia-immunodeficiency-myelokathexis syndrome (50). In this study, we show that this niche signal in human ASCs can drive activation of the ERK MAP kinase pathway and IEG expression, and that this may also be subject to fine tuning in response to potential niche signals.

Our data demonstrate that SDF1 exposure produces a pattern of gene expression in ASCs closely related to that of classical growth factor signaling (38). By contrast, in the long-term ASC culture, a distinct IEG/growth factor–like signaling module is not identified. Nevertheless, a core subset of IEGs is acutely induced but not maintained after initial exposure to conditions promoting PC maturation and survival. Thus, in ASCs, the induction of a pulse of growth factor–like signaling is induced by niche signals related to homing and survival but is rapidly attenuated and separated from gene expression associated with survival and maturation. Transient and sustained pulses of growth factor–like signaling distinguish proliferation from differentiation, promoting growth factors in models such as the PC12 neuronal cell line (29). Speculatively, the modulation of MAP kinase signal intensity and duration may also play a significant role in PC biology. In general, the importance of MAP kinase signaling in PC biology is suggested by the recurrent mutations of *NRAS*, *KRAS*, and *BRAF* in myeloma (23–25). Although the differential signaling in ASCs consequent on such mutations remains largely unexplored, a general prediction is that these impact on the kinetics and amplitude of MAP kinase pathway activation. Mutations affecting this pathway also extend to recurrent mutations in *EGR1*. Indeed, in a large recent analysis, it was shown that when EGR1 mutations occur in myeloma, these show a high clonal fraction, suggesting that the mutation either exerts a strong selective pressure or occurs as an early clonal event (5). The data presented in this study indicate that a growth factor–like signaling pathway impacting on EGR1 expression is a feature of the acute response to niche signals but is not necessarily sustained during PC survival. Furthermore, our data indicate that enhanced MAP kinase signaling can act in ASCs to sustain EGR1 protein expression. It will be interesting in future to explore what effects mutations of upstream regulators and EGR1 itself have on EGR1 behavior in PC and how these may intersect with the growth factor–like response induced by niche factors.

In conclusion, using an in vitro model system of primary human ASC differentiation and applying a gene expression networking approach, the data presented in this study argue that MAP kinase signaling and IEG regulation can be imposed by niche signals onto the ASC expression profile but are not sustained as a module integral to PC survival. ASCs can encode acute niche signals through modulation of the intensity and duration of IEG regulation, with *EGR1* providing an example of a point of niche signal integration.

## Supplementary Material

Data Supplement
