# Supplementary material for: Growth Factor–like Gene Regulation Is Separable from Survival and Maturation in Antibody-Secreting Cells
Source: J Immunol. 2019 Jan 14;202(4):1287–300. doi: 10.4049/jimmunol.1801407 (PMC6360259; doi:10.4049/jimmunol.1801407)
Supplement: Data Supplement [file JI_1801407.zip › JI_1801407_Supplemental_Figures_1.pdf]

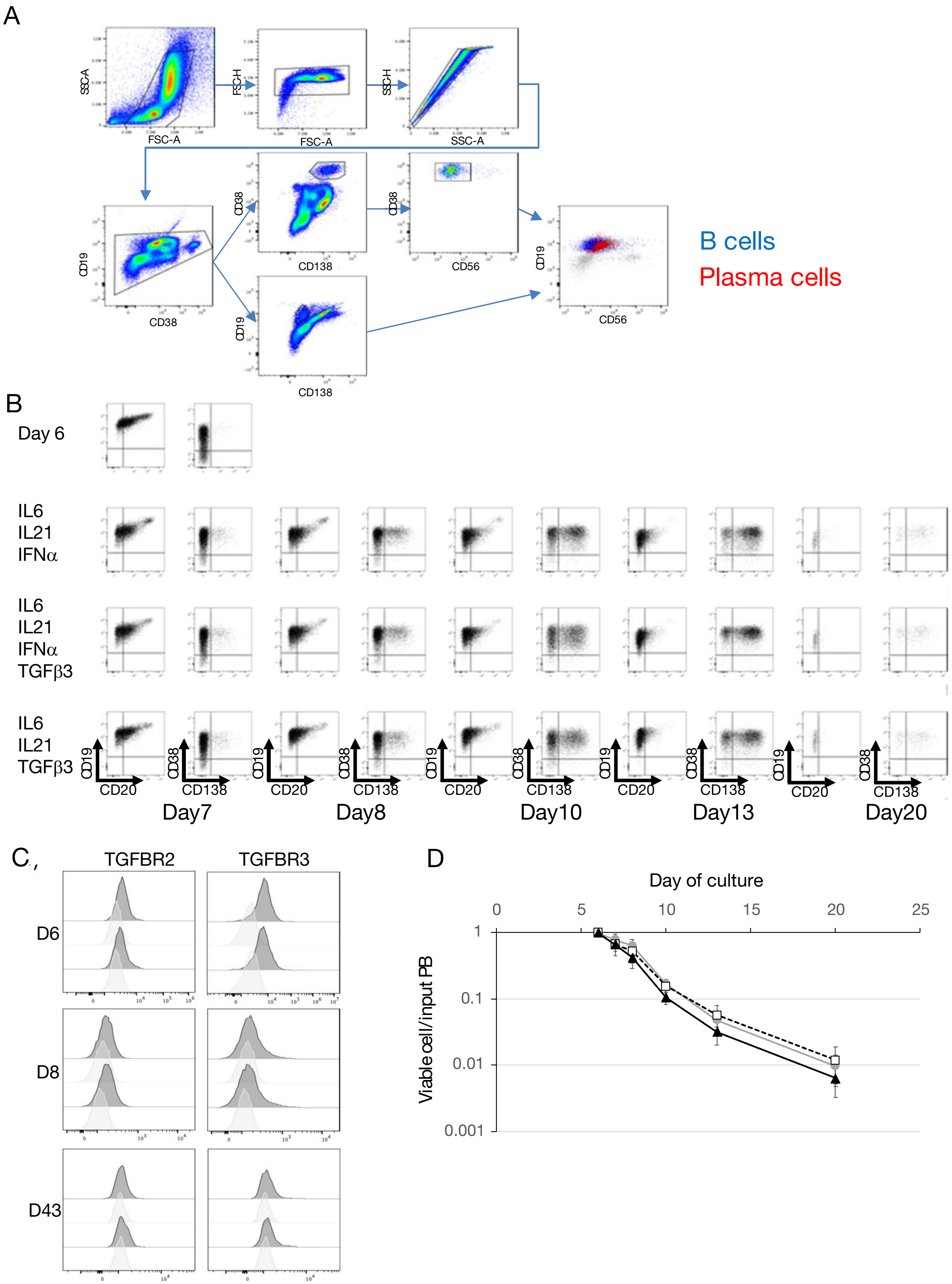


Supplemental Fig.1. Accompanies Figure 1 and 2. (A) Gating strategy for identification of bone marrow PCs to separate CD56- CD19+ PCs from B cells. (B) Phenotypic maturation of ASCs in the long-term culture under conditions used for gene expression analysis. Flow cytometry data for CD19 vs CD20 (left panel of each pair) and CD38 vs CD138 (right panel of each pair). At day 6 (top left) and each condition tested at day 7, 8, 10, 13 and 20 as indicated. (C) Flow cytometric detection of TGFBR2 (left panels) and TGFBR3 (right panels) in ASCs at day 6, day 8 and day 41 of culture, specific antibody (dark grey) isotype control (light grey) for two representative donors. (D) Viable cell numbers derived from 7AAD staining and bead-based quantification at each time point of culture shown as average and standard deviation of three donors. IL6, IL21, IFNα (grey circle and line), IL6, IL21, IFNα, TGFβ3 (open square dotted line), IL6, IL21, TGFβ3 (black triangle, solid line).

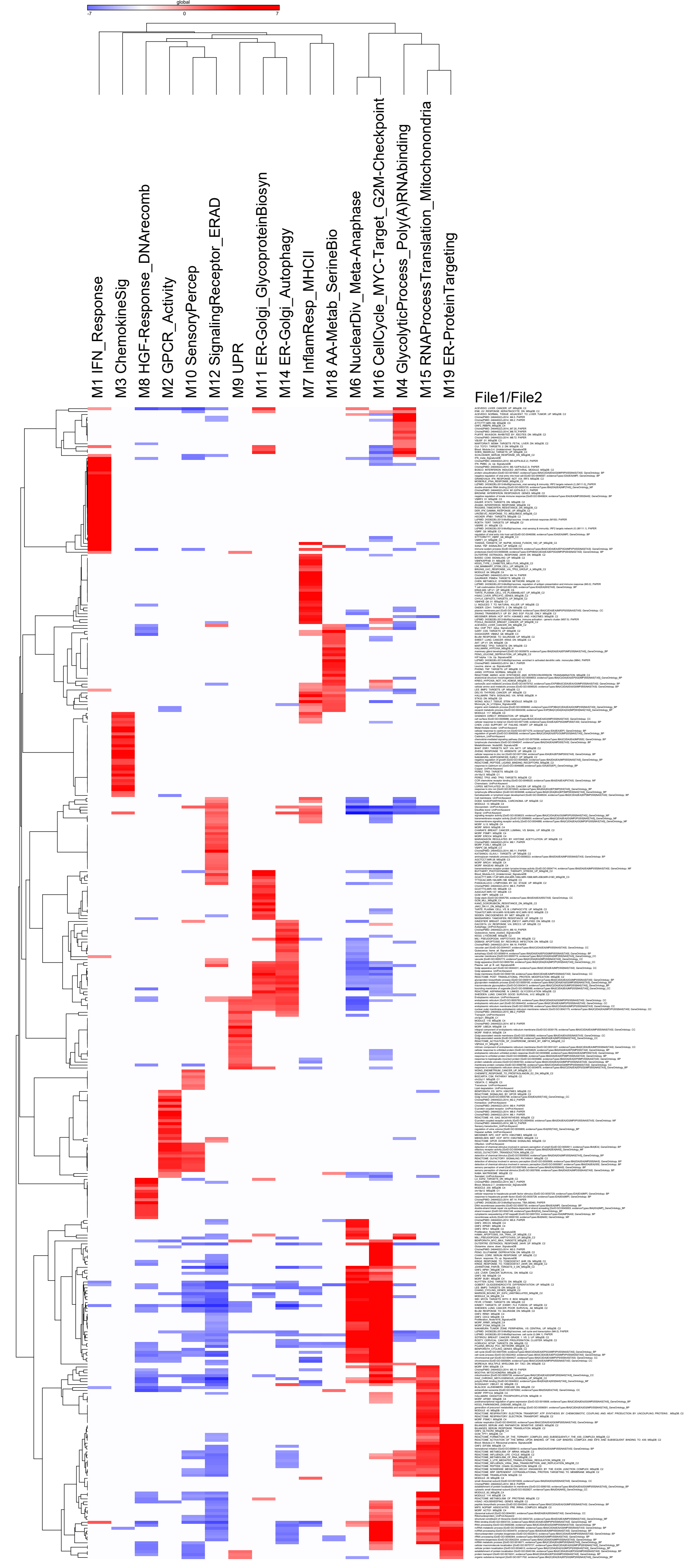


Supplemental Fig. 2. Accompanies Figure 3. High resolution version of Figure 3C. Heatmap of gene ontology and signature term enrichments linked to the PGCNA modules of the time course network analysis (filtered FDR <0.1 and ≥ 5 and ≤ 1500 genes; selecting the top 30 most significant signatures per module). For full signature enrichment lists, please see Supplemental Table 5. Modules are shown along the x-axis, and selected signature terms along the y-axis. Signature terms and modules are hierarchically clustered to illustrate relationships. Enrichment (red) and depletion (blue) of signatures are shown on color scale of z-score.

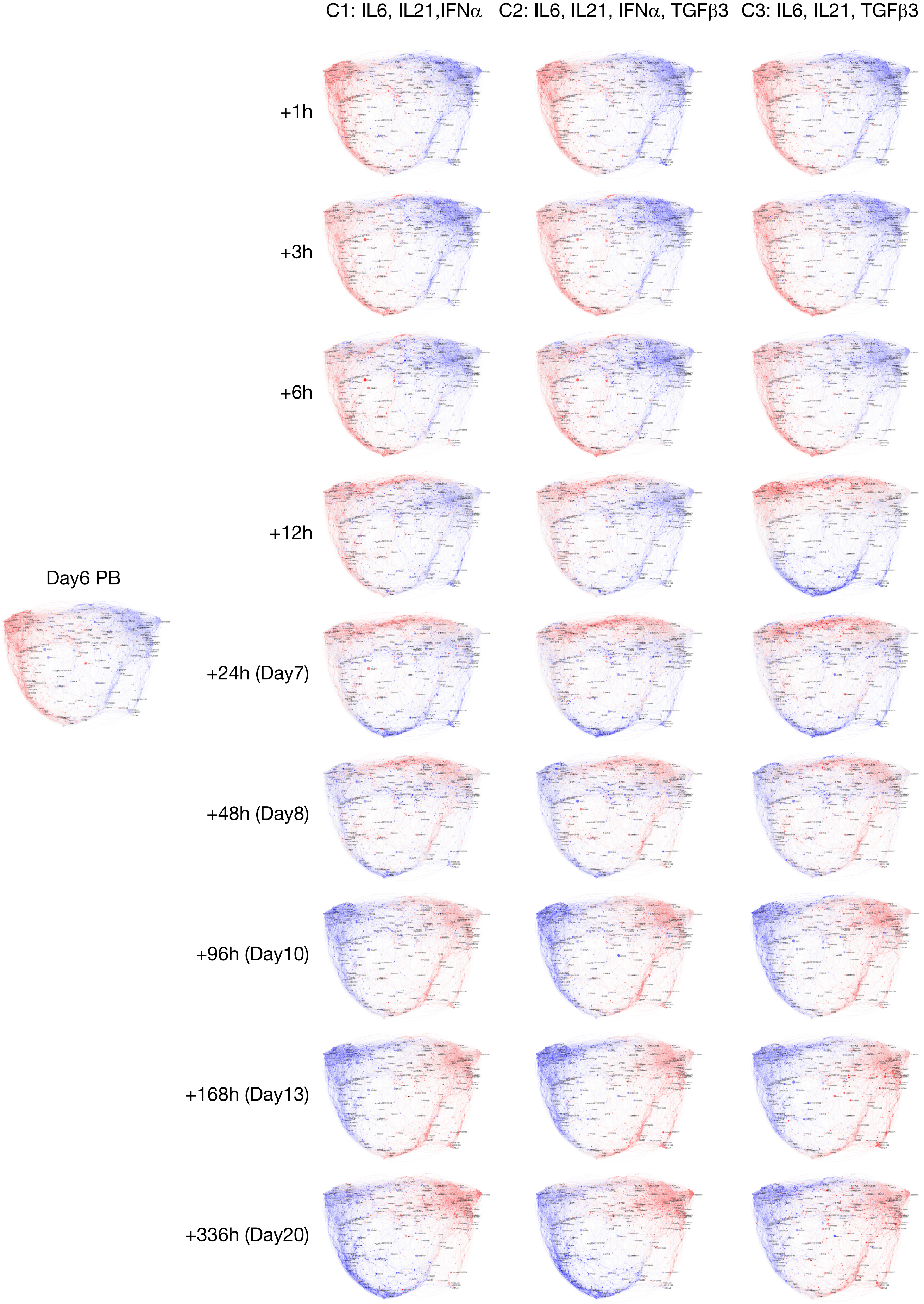


Supplemental Fig. 3. Accompanies Figure 4. Network representation of dynamics of gene expression during PB to plasma cell transition in the presence or absence of TGFβ3. Overlay of gene expression z-scores for all genes in the network shown in blue (low) to red (high) color scale. The expression state at day 6 provides the common reference with the expression patterns at each time point for each of the three conditions shown to the right. IFN α, IFNα and TGFβ3, or TGFβ3, each in the context of IL6 and IL21, shown as indicated. The time point information is illustrated across the figure. Interactive versions of all networks are available on line.

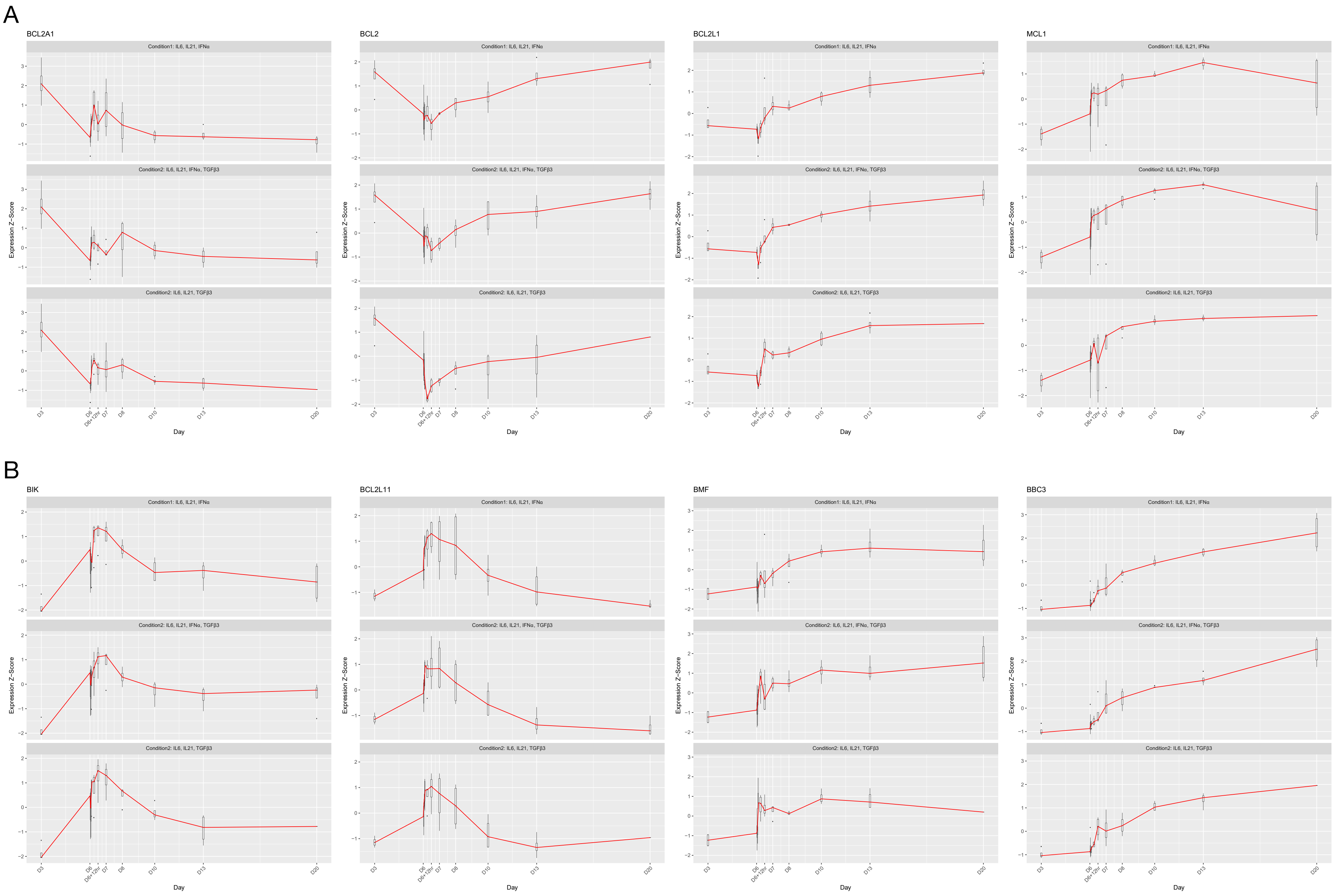


Supplemental Fig. 4. Accompanies Figure 4. Expression of BCL2 gene family members. Violin plots of gene expression for individual genes (A) anti-apoptotic gene family members BCL2A1, BCL2, BCL2L1 (BCLX) and MCL1 and (B) pro-apoptotic gene family members BIK, BCL2L11 (BIM), BMF and BBC3 (PUMA). Graphs shows gene expression z-scores (y-axis) against time in days (x-axis) and divided by the three conditions of PC differentiation upper panels (IL6, IL21, IFNα), middle panel (IL6, IL21, IFNα, TGFβ3) and lower panel (IL6, IL21, TGFβ3).

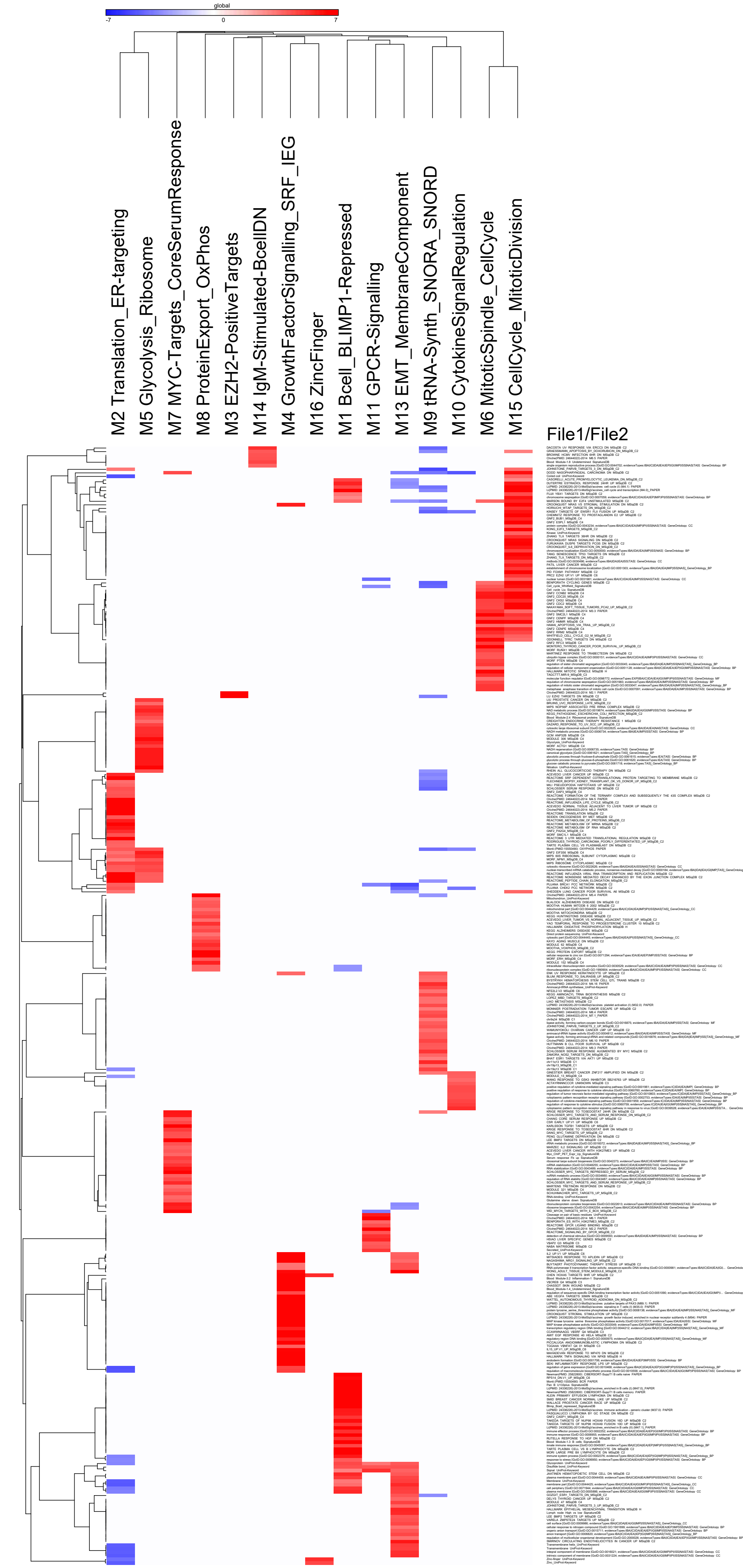


Supplemental Fig. 5. Accompanies Figure 6. Heatmap of gene ontology and signature term enrichments linked to the PGCNA modules of the SDF1 time course network analysis (filtered FDR <0.1 and ≥ 5 and ≤ 1500 genes; selecting the top 30 most significant signatures per module). For full signature enrichment lists, please see Supplemental Table 9. Modules are shown along the x-axis, and selected signature terms along the right-hand side y-axis. Signature terms and modules are hierarchically clustered to illustrate relationships. Enrichment and depletion of signatures are shown on a red to blue scale.

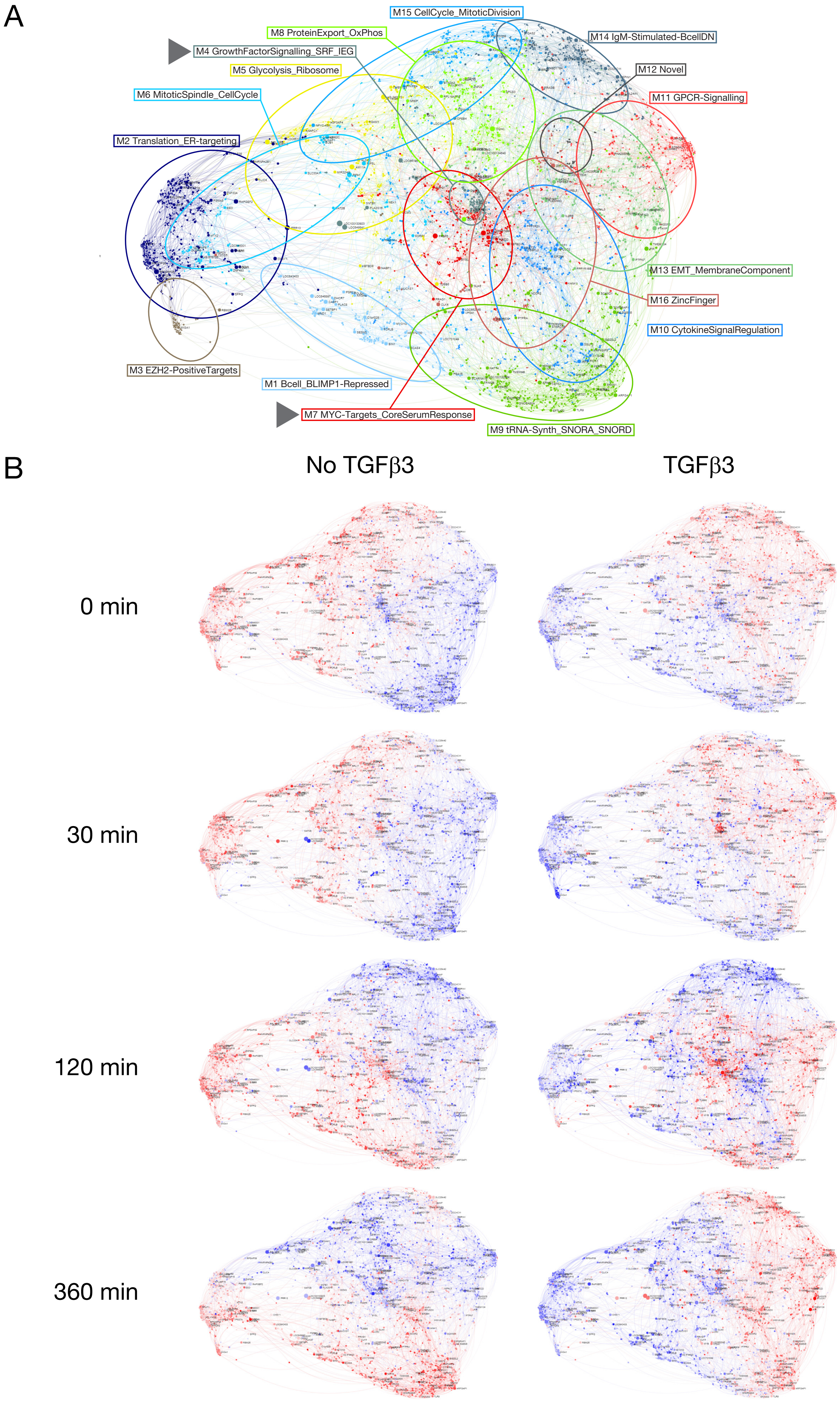


Supplemental Fig. 6. Accompanies Figure 6. (A) Network illustration with modules identified by respective summary terms. (B) Representation of dynamics of gene expression during SDF1 response in presence or absence of TGFβ3. Gene expression z-scores for all genes in the network are overlaid on network elements as blue (low) to red (high) color scale. Time points are shown on the left, with conditions no TGFβ3 left, TGFβ3 right. Annotated network with module summary terms is provided above.
